# Heart sound signals can be used for emotion recognition

**DOI:** 10.1038/s41598-019-42826-2

**Published:** 2019-04-24

**Authors:** Cheng Xiefeng, Yue Wang, Shicheng Dai, Pengjun Zhao, Qifa Liu

**Affiliations:** 1 College of Electronic and Optical Engineering, Nanjing University of Posts and Telecommunications, Nanjing, 210003 China; 2 Pediatric Cardiology, Xin Hua Hospital Affiliated to Shanghai Jiao Tong University School of Medicine, Shanghai, 200092 China; 3 College of Telecommunication and Information Engineering, Nanjing University of Posts and Telecommunications, Nanjing, 210003 China

**Keywords:** Antisense elements, Biomedical engineering

## Abstract

This article studies whether heart sound signals can be used for emotion recognition. First, we built a small emotion heart sound database, and simultaneously recorded the participants’ ECG for comparative analysis. Second, according to the characteristics of the heart sound signals, two emotion evaluation indicators were proposed: HRV of heart sounds (difference between successive heartbeats) and DSV of heart sounds (the ratio of diastolic to systolic duration variability). Then, we extracted linear and nonlinear features from two emotion evaluation indicators to recognize four kinds of emotions. Moreover, we used valence dimension, arousal dimension and valence-arousal synthesis as evaluation standards. The experimental results demonstrated that heart sound signals can be used for emotion recognition. It was more effective to achieve recognition results by combining the features of HRV and DSV of heart sounds. Finally, the average accuracy of four emotion recognitions on valence dimension, arousal dimension and valence-arousal synthesis was up to 96.875%, 88.5417% and 81.25%, respectively.

## Introduction

Emotion recognition can provide a scientific basis for monitoring of emotional health and screening for emotion-related physiology and mental disease. Emotions are not only expressed through psychological behavioral performance, but also through a series of physiological changes^1^. These physiological changes are not subjectively controlled by humans. Thus, physiological signals can more objectively reflect the true feelings of subjects^2^. Currently, many kinds of physiological signals have been successfully applied to emotion recognition, including electrocardiogram (ECG), galvanic skin response (GSR), electroencephalogram (EEG), respiratory suspended particulate (RSP) and blood volume pulse (BVP)^3–5^. For example, Jang *et al*.^6^ showed that the differences in physiological responses among emotions were significant for heart rate (HR), skin conductance level (SCL) and skin conductance response (SCR). These physiological signals, especially ECG effectively reflect the relationship between the heart beating and emotion changes. Researchers have performed much work on emotion recognition based on ECG, and heart rate variability (HRV) extracted from an ECG is now recognized as one of the important evaluation indicators of emotion recognition^7,8^.

Heart sound signals and ECG signals are different manifestations of cardiac activity. Both can effectively reflect the beating of the heart and changes in emotion^9^. Compared with the ECG signal, the acquisition process of the heart sound signal is more comfortable and convenient. Currently, ECG collection devices on the market need to directly touch the surface of the body, which may be affected by perspiration, stratum corneum and cross-infection. The shoulder-worn heart sound collector used in this paper is easy to wear and does not directly touch the body^10^; thus the above problems are avoided, and the comfort of the test is maximized. Moreover, under natural conditions, the shoulder-worn heart sound collector can collect emotion heart sound signals for a long time. In addition, an ECG can reflect the chronotropic and variable conduction of the heart but cannot reflect the inotropic ability of the heart. Heart sound signals can not only reflect the chronotropic and variable conductivity, but also reflect the inotropic ability of the heart^11^. Thus, emotion recognition based on heart sounds has irreplaceable significance.

However, whether heart sounds can be used for emotion recognition has not yet been verified by experiments; thus, this paper makes a pertinent study on heart sounds. First, we formulated an experimental plan to construct an emotion heart sound database and then simultaneously recorded the test ECG for comparison. Second, with reference to the definition of HRV and according to the characteristics of the heart sound signals, two emotion evaluation indicators were proposed as follows: heart sound HRV (HS HRV) and heart sound DSV (HS DSV). The two indicators are defined as follows:

**Definition 1**. The HS HRV (heart rate variability based on heart sound signals) is a minor difference between successive intervals of heart sound signals.

**Figure 1:**
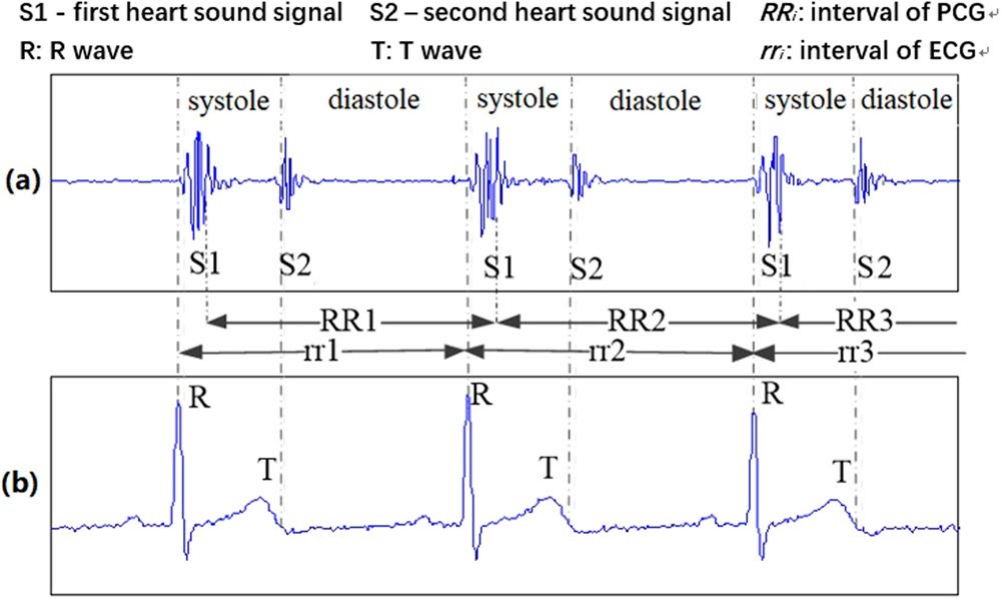
The correlation between heart sounds and ECG (**a**) heart sound signals (**b**) ECG signals.

Figure 1 shows the correlation between heart sounds and ECG. The HRV extracted from the ECG is shown in Fig. 1 as rr1, rr2, and rr3. Heart sound waveforms continuously change in S1 and S2 intervals, and the intervals of heart sound signals cannot be determined by simple peak detection. Therefore, we define intervals of heart sound as the adjacent midpoints of S1 as follows:

1 $${RR}_{i}={S}_{M}({\rm{i}}+1)-{S}_{M}({\rm{i}})$$

where $i=1,2,\,\ldots N-1$ and *N* is the number of heart sound cycles, *RR*_*i*_ is the *i*th interval of heart sound signals (shown in Fig. 1 as RR1, RR2, and RR3), *SM*(i) is the *i*th midpoint of S1, and *S*_*M*_(i + 1) is the *i* + 1 midpoint of S1.

**Definition 2**. The HS DSV (ratio of the diastolic to systolic duration variability based on heart sound signals) is a minor difference between the successive ratio of the diastolic duration to the systolic duration. In the *i*th cycle, the ratio of diastolic to systolic duration is as follows:

2 $${DS}_{i}=\frac{{D}_{i}}{{S}_{i}}$$

where $i=1,2,\mathrm{...}N-1$, *N* is the number of heart sound cycles, *D*_*i*_ is the *i*th diastolic duration and *S*_*i*_ is the *i*th systolic duration.

Extracting linear and nonlinear features from HS HRV and HS DSV achieved the emotion recognition of 4 basic emotions (relaxed, happy, sad, angry) on valence dimension, arousal dimension or valence-arousal synthesis

## Emotion Recognition System Based on Heart Sound

**Figure 2:**
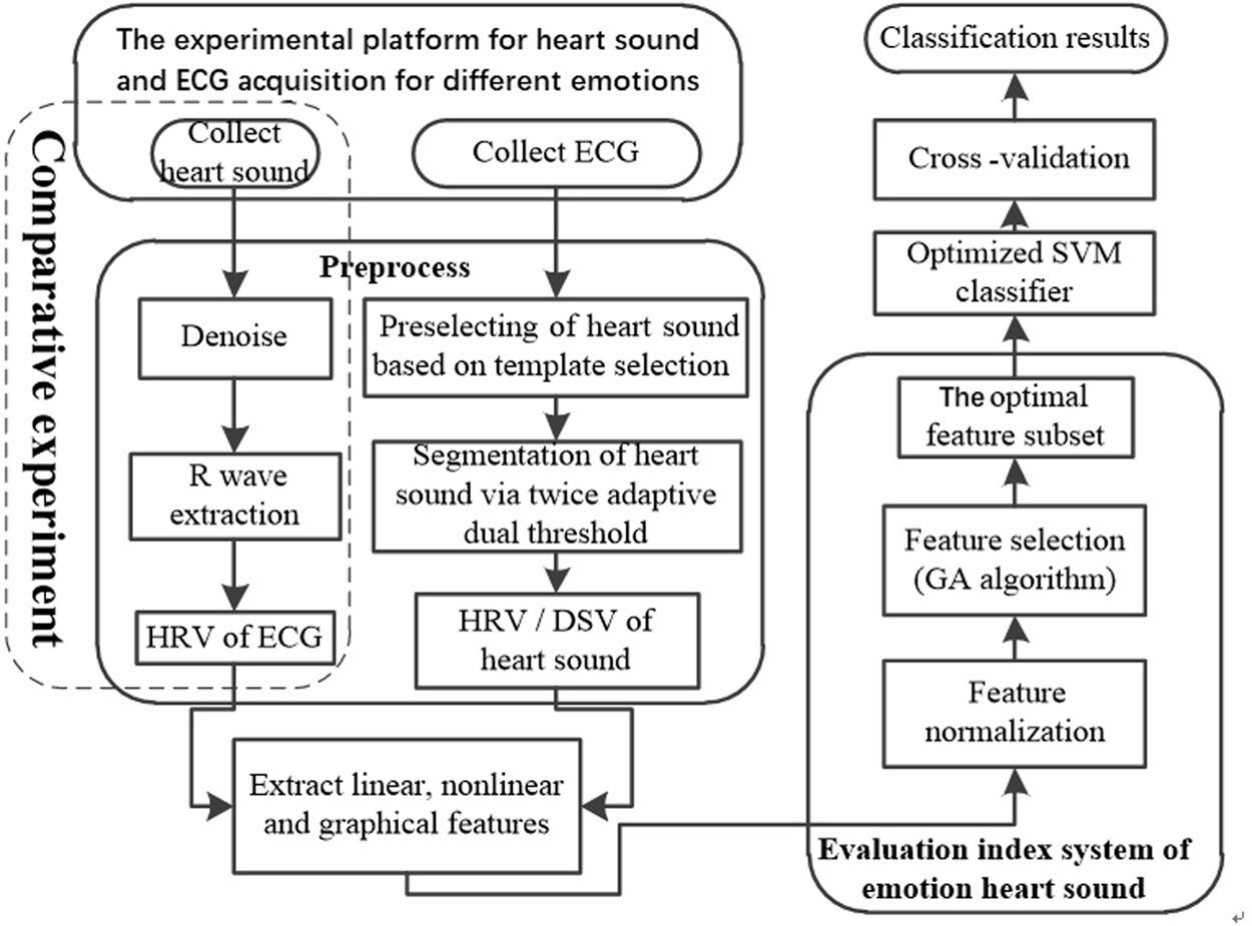
Overall block diagram of the emotion recognition system based on heart sound.

Figure 2 shows the block diagram of the emotion recognition system based on HS HRV and HS DSV. The system mainly consists of heart sound, ECG synchronous acquisition module, signal preprocessing module and emotion recognition module.

### Signal acquisition

We built shoulder-worn emotion heart sounds and ECG acquisition platforms and simultaneously collected the heart sound and ECG signals from the shoulder-worn heart sound collector and the ECG collector.

### Signal preprocessing

1. A Butterworth low pass filter was used to eliminate the background noise in heart sounds and ECG signals.
2. A heart sound preselecting and segmentation algorithm based on the template selection was used to automatically calculate HS HRV and HS DSV, using the following formula:

Segmentation results of heart sound signals were recorded in two arrays (*thb*, *tha*), and the array length was 2N (*N* is the number of heart sound cycles). In *thb*, odd numbered points represent the starting of S1, and even-numbered points represent the starting of S2. In *tha*, odd numbered points represent the ending of S1, and even-numbered points represent the ending of S2. Thus, the midpoint of S1 is expressed as *S*_*M*_(i) as follows:

3 $${S}_{M}({\rm{i}})={\rm{t}}{\rm{h}}b(2{\rm{i}}-1)+\frac{{\rm{t}}{\rm{h}}a(2{\rm{i}}-1)-{\rm{t}}{\rm{h}}{b}(2{\rm{i}}-1)}{2}$$

According to formula (1), the i th interval of heart sound is:

4 $$R{R}_{i}={S}_{M}({\rm{i}}+1)-{S}_{M}({\rm{i}})$$

According to formula (2), the i th ratio of diastolic to systolic duration is:

5 $${DS}_{i}=\frac{{\rm{t}}{\rm{h}}{b}(2{\rm{i}}+1)-{\rm{t}}{\rm{h}}{b}(2{\rm{i}})}{{\rm{t}}{\rm{h}}{b}(2{\rm{i}})-{\rm{t}}{\rm{h}}2(2{\rm{i}}-1)}$$

**Figure 3:**
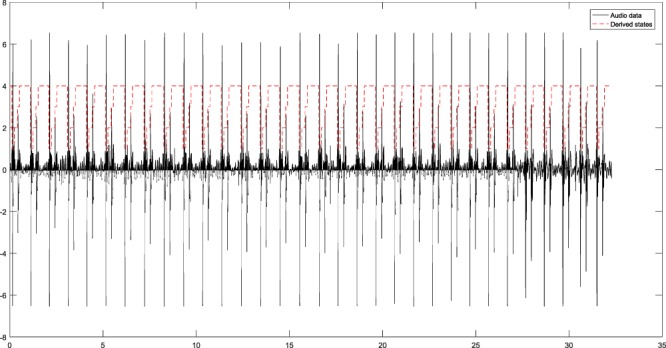
Results of the heart sound segmentation.

This paper also uses the HSMM-based heart sound segmentation algorithm proposed by Liu *et al*.^12^ to segment the heart sound signal. Compared with the segmentation algorithm based on template selection in this paper, the segmentation results are basically the same. Figure 3 shows the segment result.

Therefore, relevant data on heart sound signals can be obtained.

### Establish an emotion heart sound database

The emotion heart sound data were further processed as follows:

- Each sample of heart sounds were 300 seconds, and was divided into 2 segments; thus, 48 samples in the relaxed emotion were divided into 96 segments, 16 samples in the happy emotion were divided into 48 segments, 16 samples in the sad emotion were divided into 48 segments, 16 samples in the angry emotion were divided into 48 segments.
- Preprocessing of emotional heart sounds filtered out the data interfered by noise, such as laughter, crying and talking. Then, we accurately segmented the heart sound that passed preselecting, and only the heart sound signals with a segmentation accuracy of 100% remained in the database. In addition, to ensure the independence between each emotion heart sound, only one segment of a heart sound from the same sample eventually remained. Therefore, the emotion heart sound database eventually provided 43 segments heart sound in the relaxed emotion, 21 segments heart sound in the happy emotion, 18 segments heart sound in the sad emotion, 14 segments heart sound in the angry emotion for a total of 96 segments.
- We retained the corresponding 96 segments of the synchronized acquisition ECG signals for comparison.

### Emotion recognition

Feature extraction, feature selection and emotion recognition (cross-validation of 5-fold lines) for HS HRV, HS DSV and ECG HRV. (The features for HS HRV, HS DSV and ECG HRV are shown in Table 1 in the appendix).

**Table 1:** A comparison of the average accuracy rate of each indicator.

Signal Source	Indicator	Valence(%)	Arousal(%)	Valence-arousal Synthesis (%)
ECG	HRV	89.5833	82.2917	72.9167
Heart Sound	HRV	94.7917	90.625	80.2083
	DSV	94.7917	86.4583	78.125
	HD	96.875	88.5417	81.25

## Feature Comparison of Emotion Heart Sound and ECG

**Figure 4:**
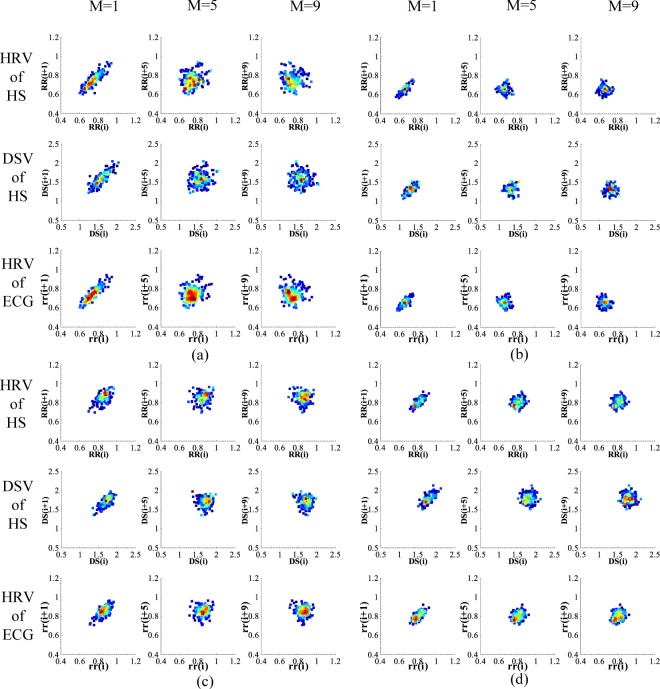
The LPP for HS HRV, HS DSV and ECG HRV for different emotions. Relaxed (**b**) Happy (**c**) Sad (**d**) Angry.

After extracting linear and nonlinear features from HS HRV, HS DSV and ECG HRV in the 96 segments emotion heart sound signals. According to the numerical distribution of various features and the distribution of figures, the representation results of three emotion evaluation indicators were different, but the overall representation results were consistent. Using the Lagged Poincaré Plot (LPP) as an example, Fig. 4 shows the LPP for HS HRV, HS DSV and ECG HRV for different emotions. Figure 4(a) shows the comparison of the LPP under the three indicators for the relaxed emotion. The points in the figure are evenly distributed and noticeably change with the lag dimension M. Figure 4(b) shows the comparison of the LPP for the three indicators for the happy emotion. The points under the figure are densely distributed and noticeably change with the lag dimension M; Fig. 4(c) shows the comparison of the LPP for the three indicators for the sad emotion. The points in the figure are evenly distributed, are mainly focused on the middle section, and change slowly with the lag dimension M; Fig. 4(d) shows the comparison of the LPP for the three indicators for the angry emotion. The points in the figure are the most concentrated, and change slowly with the lag dimension M.

A previous study^5^ conducted on emotion recognition based on ECG HRV determined that in ECG HRV in LPP, the points are evenly distributed in the low arousal dimensions (relaxed and sad emotion states), and the points are densely distributed in the high arousal dimension (happy and angry emotion states). This result was consistent with the regularity of HS HRV, HS DSV and ECG HRV in this paper, indicating that the emotions of the subjects were fully stimulated when heart sound signals and ECG signals were recorded. The HS HRV and HS DSV are effective emotion evaluation indicators, and heart sounds can be used for emotion recognition. In addition, another previous study^13^ concluded that HRV indices showed significant differences between happy and sad emotion states.

Features extracted from the LPP contain rich information, and some of the figure features extracted from HS DSV had more effective representation than HS HRV and ECG HRV. For example, feature SD12 (The ratio between SD1 and SD2) as follows:

6 $$SD12=\frac{SD1}{SD2}$$

SD1 represents the length of the short half axis of the LPP fitted with an ellipse (the major axis of the ellipse extends along a 45° direction), and SD2 represents the length of the long half axis of the LPP.

7 $$\{\begin{array}{c}SD1=\sqrt{\frac{1}{N-M}\sum _{i=1}^{N-M}\frac{{({RR}_{i}-{RR}_{i+M})}^{2}}{2}}\\ SD2=\sqrt{\frac{1}{N-M}\sum _{i=1}^{N-M}\frac{{({RR}_{i}+{RR}_{i+M}-2\overline{RR})}^{2}}{2}}\end{array}$$

**Figure 5:**
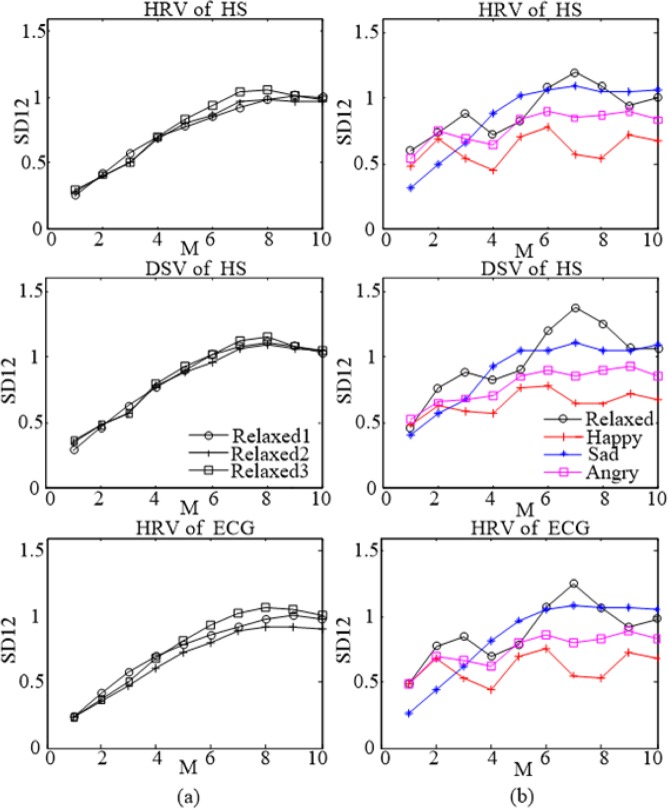
Feature SD12 changes with lag dimension M during the sessions. (**a**) SD12 of HS HRV, HS DSV and ECG HRV from the same subject during 3 sessions in the relaxed emotion state. (**b**) SD12 of HS HRV, HS DSV and ECG HRV from the same subject during 4 sessions in different emotions.

Figure 5 shows that SD12 changes with lag dimension M during the sessions. Figure 5(a) shows SD12 curves of HS HRV, HS DSV and ECG HRV from the same subject during 3 sessions in the relaxed emotion state. The distribution of SD12 curves of the three indicators has similarities during three sessions in a relaxed emotion state; however, compared with the other two indicators, the HS DSV is more concentrated. Thus, the HS DSV has a more noticeable representation of the same emotion. Figure 5(b) shows the SD12 of HS HRV, HS DSV and ECG HRV from the same subject during 4 sessions in different emotions.

During 4 sessions, SD12 curves with low arousal dimension (relaxed and sad emotion states) are higher than SD12 curves with high arousal dimension (happy and anger emotion states), and HS DSV is more clearly separated than the other two indicators. Thus, HS DSV is more notable for the representation of different emotions.

A previous study^5^ found that with the increase of arousal, the degree of separation between the arousal emotion and corresponding neutral emotion increases. This was consistent with the SD12 of HS HRV, HS DSV and ECG HRV in this paper. In high emotional arousal dimensions (happy and angry emotion states), the SD12 is lower; however, in low emotional arousal dimensions (relaxed and sad emotion states), the SD12 is higher. This result showed that emotions of the subjects were fully stimulated when heart sound signals and ECG signals were recorded, proving that HS HRV and HS DSV are effective emotion evaluation indicators and that heart sounds can be used for emotion recognition.

More feature comparisons of emotion heart sound and ECG are shown in the appendix.

## Experiment Platform

**Figure 6:**
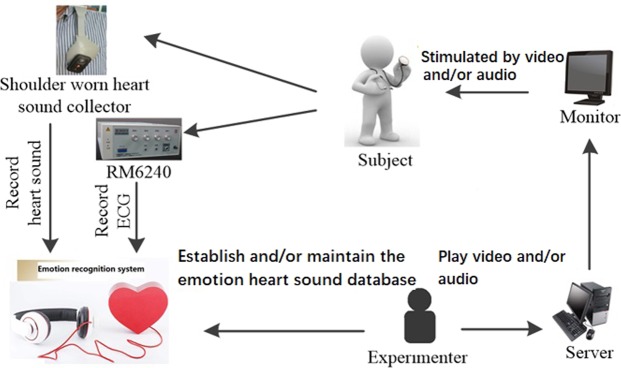
Emotion heart sounds and ECG acquisition platform.

Shoulder-worn emotion heart sounds and ECG acquisition platforms are shown in Fig. 6. The hardware devices of the platforms included shoulder-worn heart sound collector, an ECG signal collector, an RM6240 multichannel biosignal recorder, two servers, three monitors, a pair of headphones and a mouse.

The shoulder-worn heart sound collector is an Ω-type wearable device for collecting human heart sound signals. The device is made of lightweight elastic material and is formed as an Ω-shaped frame, which is similar to the outline of the human shoulder to chest. Thus, the top of the Ω-shaped frame can be conveniently placed on the left shoulder, and the heart sound sensor can be in a fixed position on the chest end. In addition, the elastic pressure generated by the Ω-shaped frame elastic material can make the heart sound sensor close to the apex of the human body to obtain the best heart sound^10^.

The software system, including preprocessing, feature extraction, feature selection, recognition of heart sound and ECG signals, was written by the author using MATLAB 2012a.

Sixteen (12 males and six females) healthy, outgoing and optimistic college students and graduate students aged 18 to 26 participated in the experiment. All participants passed the Eysenck Personality Test in advance, and their spirits were normal. The Self-reports from volunteers showed that they had no history of hearing and visual impairments, and they did not suffer from any cardiovascular or chronic diseases. The heart sound collection experiment on volunteers was approved by the Biological and Medical Ethics Committee of the Dalian University of Technology. The volunteers were informed on the content, the purpose and the precautions of the experiment, and informed consent was signed in advance. However, volunteers were not informed what kind of materials they would hear and/or see. Volunteers remained static during the experiment to avoid affecting the ECG collection. To reduce the interference of external sounds on heart sound signals, sound signals were received via headphones.

**Figure 7:**
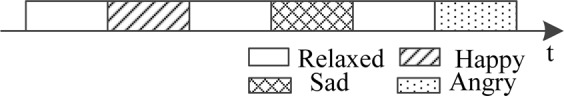
Timeline for different emotions.

In this paper, we selected video and music materials to stimulate different emotions. The stimuli material for the relaxed emotion states was music, and the stimuli material for the rest of the emotions were videos. Emotion-stimuli material consisted of 6 sessions. The timeline is shown in Fig. 7, and the white squares, slashed squares, grid squares and dotted squares represent relaxed, happy, sad and angry emotions respectively. After a sufficient amount of rest, according to the timeline, volunteers were scheduled to watch videos or to listen to music. All audios and videos were selected from international affective picture system (IAPS), international affective digitized sounds (IADS) and Chinese affective digital sounds. The relaxed emotion state was stimulation with light music for 4 minutes and 30 seconds; The happy emotion state was stimulation with the variety show called “The Ellen Show” for 6 minutes and 33 seconds; The sad emotion state was stimulation with a movie clip of the Tangshan earthquake for 3 minutes and 28 seconds and stimulation with the public service advertising called “A Father’s journey” for 5 minutes and 4 seconds; The angry emotion state was stimulation with the movie clip of “Tokyo Trial” for 6 minutes and 58 seconds. After each session, volunteers were required to complete a feedback questionnaire, and their heart sound and ECG signals were recorded throughout the experiment.

## Recognition Results and Analysis

According to the HS HRV, HS DSV, ECG HRV and HRV and DSV of heart sound (HS HD), we created 79-dimensional, 75-dimensional, 79-dimensional,154-dimensional original feature matrixes. Then, we used genetic algorithm (GA) to optimize the SVM classifier to select the optimal feature subset for emotion recognition. The emotion recognition system recognizes emotions on the valence dimension, emotional arousal and valence-arousal synthesis. The results are in the sections below:

### On valence dimension

**Figure 8:**
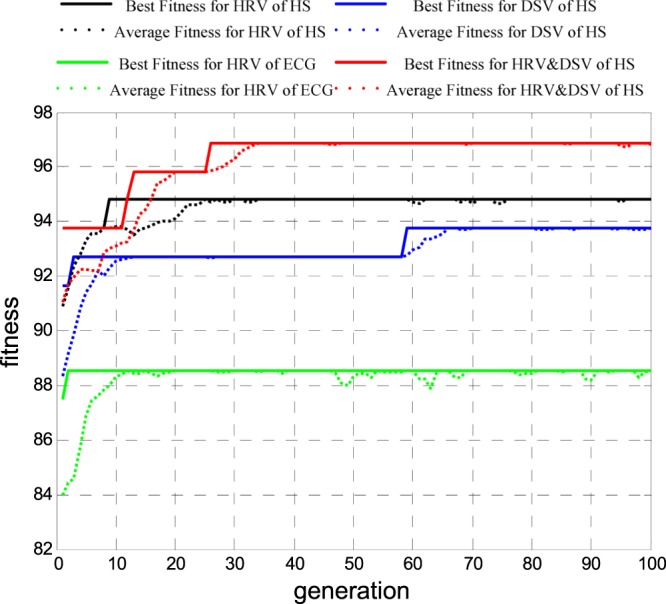
Adaptation function evolution curve of each indicator when selecting features for emotion recognition on different valence dimensions by GA.

According to Russell’s circumplex model of emotions^14^, sad and angry emotions are in the low valence dimension, while relaxed and happy emotions are on high valence dimension; thus, 32 samples were on the low valence, 64 samples were on the high valence. The GA optimized SVM classifier was used for feature selection. To reduce the contingency of selecting the optimal feature subsets, we repeated the feature selection operations 30 times. Figure 8 shows the adaptation function evolution curve of each indicator when selecting features for emotion recognition on different valence dimensions by GA. The solid line is the best fitness, and the dotted line is the average fitness. Lines in black, blue, green and red are HS HRV, HS DSV, ECG HRV and HS HD. With the evolution of feature subsets, the classification accuracy rate was continuously improved and tended to be stable. The searched highest recognition rate corresponded to the optimal feature subset of the experiment. If this recognition rate ranked in the top 20% in 30 iterations, this feature subset was involved in the calculation of the weight coefficient, which determined the optimal feature subset for valence recognition.

**Figure 9:**
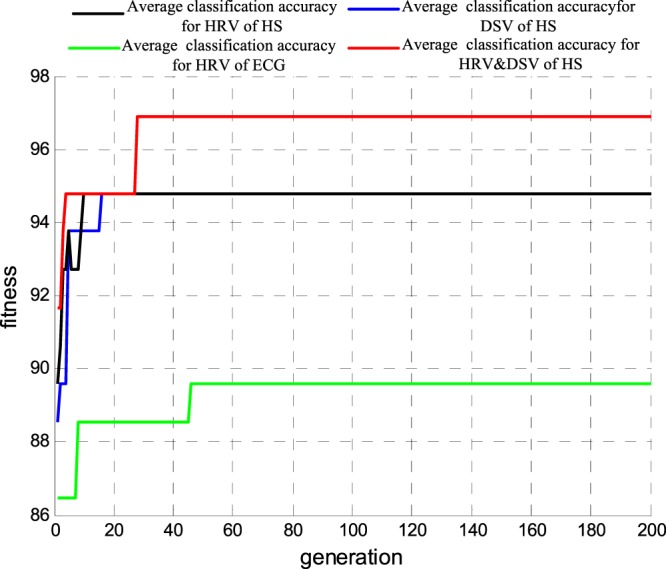
The average accuracy rate curve of each indicator for different valence emotion recognition.

Finally, using the optimal feature subset as the input of the optimized SVM classifier, we obtained results from emotional recognition on different valences. In Fig. 9, lines in black, blue, green and red correlate with the average accuracy rate (cross-validation of 5-fold lines) for HS HRV, HS DSV, ECG HRV and HS HD. With the evolution of parameters c and g, the average accuracy rate was continuously improved and tended to be stable. The highest accuracy rate corresponding to the parameters c and g constituted the best valence recognition model.

### On emotional arousal

**Figure 10:**
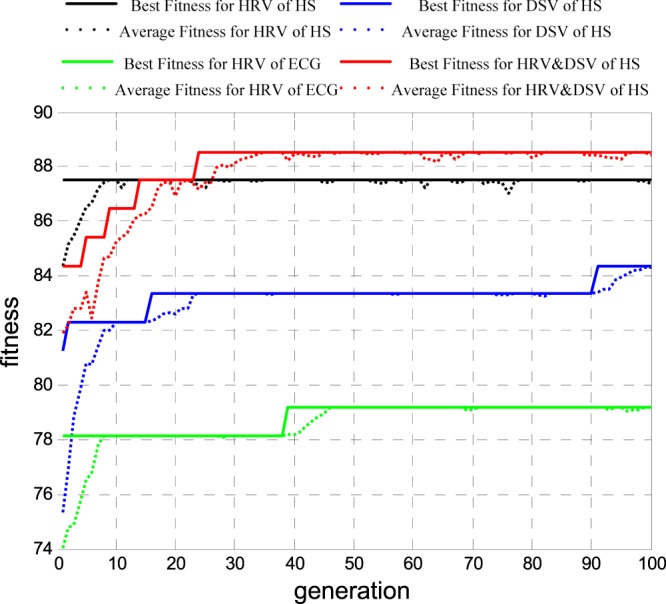
Adaptation function evolution curve of each indicator when selecting features for emotion recognition on different arousal dimensions by GA.

According to Russell’s circumplex model of emotions^14^, relaxed and sad emotion states are in the low emotional arousal dimension, while happy and angry emotion states are in the high emotional arousal dimension. Thus, 61 samples were on low arousal dimension, 35 samples were on high arousal dimension. The GA optimized SVM classifier was used for feature selection. The feature selection operations were repeated 30 times. In one of these operations, Fig. 10 shows the adaptation function evolution curve of each indicator when selecting features for emotion recognition on different emotional arousal by GA; the solid line is the best fitness, and the dotted line is the average fitness. Lines in black, blue, green and red correspond to HS HRV, HS DSV, ECG HRV and HS HD, respectively. With the evolution of feature subsets, the classification accuracy rate was continuously improved and tended to be stable. The searched highest recognition rate corresponded to the optimal feature subset of the experiment. If this recognition rate ranks in the top 20% in 30 iterations, this feature subset was involved in the calculation of the weight coefficient, which determined the optimal feature subset for arousal recognition.

**Figure 11:**
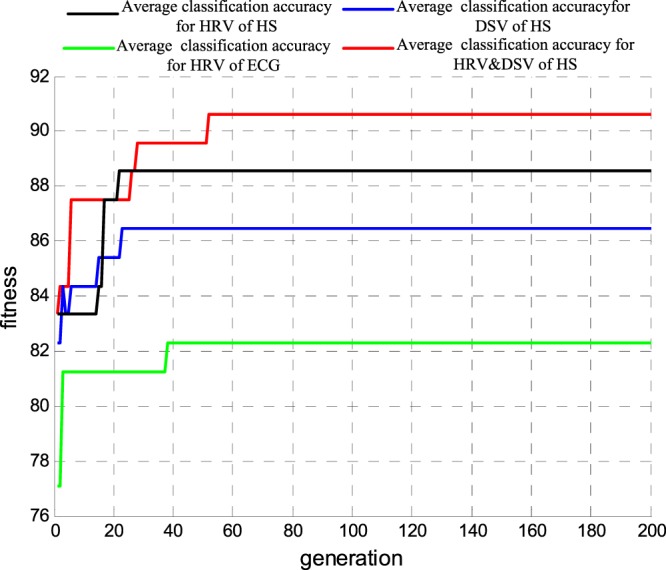
The average accuracy rate curve of each indicator for different arousal emotion recognition.

Finally, using the optimal feature subset as the input of the optimized SVM classifier, we obtained the results of the emotion recognition on different arousal states. In Fig. 11, lines in black, blue, green and red represent the average accuracy rate (cross-validation of 5-fold lines) for HS HRV, HS DSV, ECG HRV and HS HD, respectively. With the evolution of parameters c and g, the average accuracy rate was continuously improved and tended to be stable. The highest accuracy rate corresponded to the parameters c and g constituted the best arousal recognition model.

### Valence-arousal synthesis

**Figure 12:**
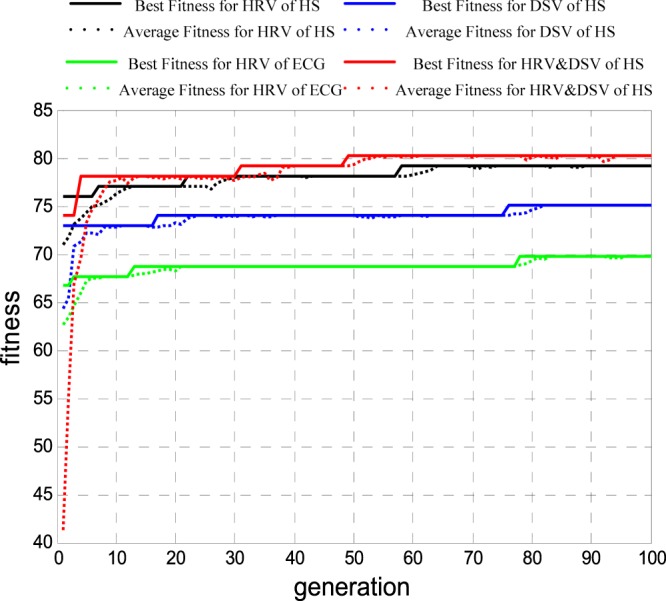
Adaptation function evolution curve of each indicator when selecting features for emotion recognition on valence-arousal synthesis by GA.

According to Russell’s circumplex model of emotions^14^, four emotions (relaxed, happy, sad, angry) are distributed in different quadrants of the model; thus, this model is a kind of valence-arousal synthesis recognition system. There were 43, 21, 18, and 14 samples in the four emotions of relaxed, happy, sad and angry, respectively. In one of these operations, Fig. 12 shows the adaptation function evolution curve of each indicator when selecting features for emotion recognition on valence-arousal synthesis by GA; the solid line is the best fitness, and the dotted line is the average fitness. Lines in black, blue, green and red correspond to HS HRV, HS DSV, ECG HRV and HS HD, respectively. With the evolution of feature subsets, the classification accuracy rate was continuously improved and tended to be stable. The searched highest recognition rate corresponded to the optimal feature subset of the experiment. If this recognition rate ranked in the top 20% in 30 iterations, this feature subset was involved in the calculation of the weight coefficient, which determined the optimal feature subset for valence-arousal synthesis recognition.

**Figure 13:**
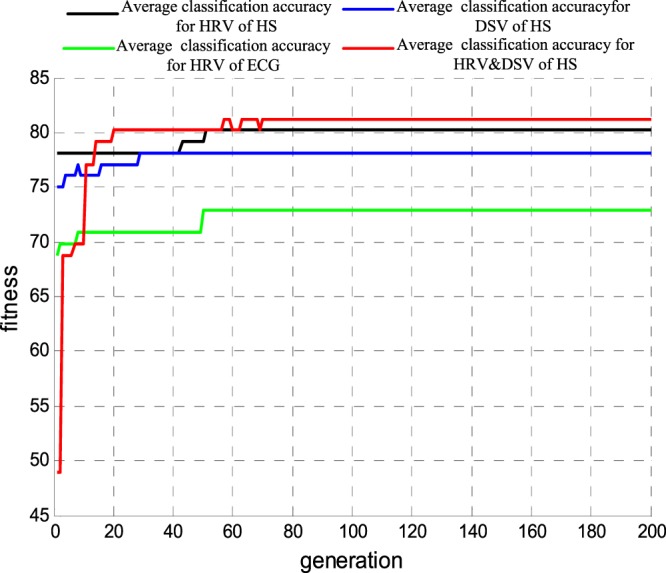
The average accuracy rate curve of each indicator for 4 kinds of emotions recognition.

Finally, using the optimal feature subset as the input of the optimized SVM classifier, we obtained the results of the emotion recognition for 4 kinds of emotions. In Fig. 13, lines in black, blue, green and red represent the average accuracy rate (cross-validation of 5-fold lines) for HS HRV, HS DSV, ECG HRV and HS HD. With the evolution of parameters c and g, the average accuracy rate was continuously improved and tended to be stable. The highest accuracy rate corresponded to the parameters c and g constituted the best valence-arousal synthesis model.

## Conclusions

The performance of four kinds of emotion recognition indicators on valence dimension, emotional arousal and valence-arousal synthesis, the average accuracy rate of each indicator was compared and is shown in Table 1.

As shown in Table 1, (1) the three indicators have noticeable advantages in the recognition of emotions on different valence dimensions, and the average accuracy rate in different valence dimensions was higher than that in different emotional arousal and valence-arousal syntheses. (2) The results were not a simple linear relationship between the features of indicators; it is impossible to divide different emotions in different quadrants, as shown in Russell’s circumplex model of emotions. Thus, the average accuracy rate in valence-arousal synthesis was lower than that in valence dimension and emotional arousal. (3) According to the experimental results, the specificity and sensitivity of the performed classification is 0.9796 and 0.9932, respectively. (4) Heart sound signals can be used for emotion recognition, and HS HRV and HS DSV were effective emotion evaluation indicators. The average accuracy rate for HS HRV was higher than HS DSV and ECG HRV. In the selection of experimental data, we all selected the noticeable part of the heart sound signal during the process of the emotion change and then compared this heart sound signal with the ECG signal from the same segment. This result may be the main reason for the higher accuracy in the heart sound than in the ECG.

Although the average accuracy rate for the HS DSV was lower than that for the HS HRV, when combining the features of HRV and DSV together to recognize emotions, the average accuracy rate was higher than two indicators alone. This result showed that the HS HD had the best representation results, and the proposal of the DSV indicator had an important role, while traditional ECG signals were not achieved.

In summary, (1) heart sound signals can be used for emotion recognition; (2) combining the features of HRV and DSV together can achieve better recognition results, and the proposal of the DSV indicator has an important mean.

## Supplementary information

appendix of the paper
