# Supplementary material for: Heart sound signals can be used for emotion recognition
Source: Sci Rep. 2019 Apr 24;9:6486. doi: 10.1038/s41598-019-42826-2 (PMC6482302; doi:10.1038/s41598-019-42826-2)
Supplement: Supplementary file 1 — appendix of the paper [file 41598_2019_42826_MOESM1_ESM.docx]

Heart sound signals can be used for emotion recognition

Cheng Xiefeng ^^[[1]](#footnote-1)^^ Yue Wang ^1^ Shicheng Dai ^1^ Pengjun Zhao ^2^ Qifa Liu^1,3^

^1 College of Electronic and Optical Engineering, Nanjing University of Posts and Telecommunications, Nanjing,210003^

^2 Pediatric Cardiology, Xin Hua Hospital Affiliated to Shanghai Jiao Tong University School of Medicine, Shanghai,^ [^200092^](http://www.youbian.com/200092/)

^3 College of Telecommunication and Information Engineering, Nanjing University of Posts and Telecommunications, Nanjing,210003^

^Correspondence to [dennisdsc@163.com]^

**Appendix**

**Other features comparison of emotion heart sound and ECG**

The 41 features for HS HRV form a 79-dimensional original feature matrix; The 37 features for HS DSV form a 75-dimensional original feature matrix; The 41 features for HRV of ECG form a 79-dimensional original feature matrix and combining the features of HS HRV and HS DSV together form a 154-dimensional original feature matrix. Specific features are showed in Appendix Table 1. All 154-dimensional features of the heart sound signal are split into two parts recorded in the table, but 154-dimensional features are used integratedly.

Appendix Table 1 Feature list for HS HRV, HS DSV, HRV of ECG

| No. | HS HRV | HS DSV | HRV of ECG | HRV of HD | DSV of HD |
| --- | --- | --- | --- | --- | --- |
| 1 | $RRmean$ | $DSmean$ | $rrmean$ | $RRmean$ | $DSmean$ |
| 2 | $RRmedian$ | $DSmedian$ | $rrmedian$ | $RRmedian$ | $DSmedian$ |
| 3 | HRmean | Meaningless | HRmean | HRmean | Meaningless |
| 4 | HRstd | Meaningless | HRstd | HRstd | Meaningless |
| 5 | SDRR | $SDDS$ | SDrr | SDRR | $SDDS$ |
| 6 | SDSD | SDSD | SDSD | SDSD | SDSD |
| 7 | RMSSD | RMSSD | RMSSD | RMSSD | RMSSD |
| 8 | RR50 | Meaningless | rr50 | RR50 | Meaningless |
| 9 | pRR50 | Meaningless | prr50 | pRR50 | Meaningless |
| 10 | HS HRV triangular index | DSV triangular index | HRV of ECG triangular index | HS HRV triangular index | DSV triangular index |
| 11 | TIRR | TIDS | TIrr | TIRR | TIDS |
| 12 | Ultra low frequency energy aVLF | | | | |
| 13 | low frequency energy aLF | | | | |
| 14 | high frequency energy aHF | | | | |
| 15 | Total energy aTotal | | | | |
| 16 | Low frequency energy standard value nLF | | | | |
| 17 | High frequency energy standard value nHF | | | | |
| 18 | Low frequency/high frequency energy ratioLF/HF | | | | |
| 19 | Ultra-low frequency peak peakVLF | | | | |
| 20 | Low frequency peak peakLF | | | | |
| 21 | high frequency peak peakHF | | | | |
| 22 | Lyapunov index Lamb | | | | |
| 23 | Approximate entropy ApEn | | | | |
| 24 | Sample entropy SampEn | | | | |
| 25 | DFA in short-term$\alpha1$ | | | | |
| 26 | DFA in long-term $\alpha2$ | | | | |
| 27 | Ellipse area when M=1 S1 | | | | |
| 28 | Ellipse area when M=2 S2 | | | | |
| 29 | Ellipse area when M=3 S3 | | | | |
| 30 | Ellipse area when M=4 S4 | | | | |
| 31 | Ellipse area when M=5 S5 | | | | |
| 32 | Ellipse area when M=6 S6 | | | | |
| 33 | Ellipse area when M=7 S7 | | | | |
| 34 | Ellipse area when M=8 S8 | | | | |
| 35 | Ellipse area when M=9 S9 | | | | |
| 36 | Ellipse area when M=10 S10 | | | | |
| 37 | Short half shaft length when M=1 1SD1 | | | | |
| 38 | Short half shaft length when M=2 2SD1 | | | | |
| 39 | Short half shaft length when M=3 3SD1 | | | | |
| 40 | Short half shaft length when M=4 4SD1 | | | | |
| 41 | Short half shaft length when M=5 5SD1 | | | | |
| 42 | Short half shaft length when M=6 6SD1 | | | | |
| 43 | Short half shaft length when M=7 7SD1 | | | | |
| 44 | Short half shaft length when M=8 8SD1 | | | | |
| 45 | Short half shaft length when M=9 9SD1 | | | | |
| 46 | Short half shaft length when M=10 10SD1 | | | | |
| 47 | long half shaft length when M=1 1SD2 | | | | |
| 48 | long half shaft length when M=2 2SD2 | | | | |
| 49 | long half shaft length when M=3 3SD2 | | | | |
| 50 | long half shaft length when M=4 4SD2 | | | | |
| 51 | long half shaft length when M=5 5SD2 | | | | |
| 52 | long half shaft length when M=6 6SD2 | | | | |
| 53 | long half shaft length when M=7 7SD2 | | | | |
| 54 | long half shaft length when M=8 8SD2 | | | | |
| 55 | long half shaft length when M=9 9SD2 | | | | |
| 56 | long half shaft length when M=10 10SD2 | | | | |
| 57 | Ratio between SD1 and SD2 when M=1 1SD12 | | | | |
| 58 | Ratio between SD1 and SD2 when M=2 2SD12 | | | | |
| 59 | Ratio between SD1 and SD2 when M=3 3SD12 | | | | |
| 60 | Ratio between SD1 and SD2 when M=4 4SD12 | | | | |
| 61 | Ratio between SD1 and SD2 when M=5 5SD12 | | | | |
| 62 | Ratio between SD1 and SD2 when M=6 6SD12 | | | | |
| 63 | Ratio between SD1 and SD2 when M=7 7SD12 | | | | |
| 64 | Ratio between SD1 and SD2 when M=8 8SD12 | | | | |
| 65 | Ratio between SD1 and SD2 when M=9 9SD12 | | | | |
| 66 | Ratio between SD1 and SD2 when M=10 10SD12 | | | | |
| 67 | Box dimension of recursive plot Box dimension | | | | |
| 68 | Generalized dimension of recursive plot D1 | | | | |
| 69 | Generalized dimension of recursive plot D2 | | | | |
| 70 | Recurrence rate | | | | |
| 71 | Determinism | | | | |
| 72 | Averaged diagonal length | | | | |
| 73 | Length of longest diagonal line | | | | |
| 74 | Entropy of diagonal length | | | | |
| 75 | Laminarity | | | | |
| 76 | Trapping time | | | | |
| 77 | Length of longest vertical line | | | | |
| 78 | Recurrence time of 1st type | | | | |
| 79 | Recurrence time of 2nd type | | | | |

**Typical feature——Mean**

Appendix Table 2 the feature Mean comparison for HS HRV, HS DSV and HRV of ECG in four emotions

| Features | Relaxed | | Happy | | Sad | | Angry | |
| --- | --- | --- | --- | --- | --- | --- | --- | --- |
|  | average | std | average | std | average | std | average | std |
| RRmean/ms | 816.1233 | 87.9764 | 781.6381 | 119.4929 | 798.9167 | 68.42098 | 778.8857 | 62.72259 |
| DSmean | 1.68578 | 0.20867 | 1.61026 | 0.21913 | 1.67818 | 0.16651 | 1.64809 | 0.18055 |
| rrmean | 810.7512 | 77.23445 | 781.2524 | 119.6518 | 798.1056 | 69.82536 | 778.1571 | 62.53196 |

**Typical feature——Triangular index**

Appendix Table 3 the feature Triangular index comparison for HS HRV, HS DSV and HRV of ECG in four emotions

| Features | Relaxed | | Happy | | Sad | | Angry | |
| --- | --- | --- | --- | --- | --- | --- | --- | --- |
|  | average | std | average | std | average | std | average | std |
| HS HRV triangular index | 11.36279 | 1.749425 | 10.09524 | 1.537685 | 12.15 | 1.729077 | 11.55714 | 1.811805 |
| DSV triangular index | 11.14419 | 1.521638 | 9.690476 | 1.181061 | 11.47222 | 1.569865 | 10 | 1.36043 |
| HRV of ECG triangular index | 9.351163 | 2.195707 | 9.509524 | 2.037622 | 9.666667 | 1.388185 | 9.557143 | 2.174225 |

**Typical feature——Approximate triangle base width**

Appendix Table 4 the feature Approximate triangle base width comparison for HS HRV, HS DSV and HRV of ECG in four emotions

| Features | Relaxed | | Happy | | Sad | | Angry | |
| --- | --- | --- | --- | --- | --- | --- | --- | --- |
|  | average | std | average | std | average | std | average | std |
| TIRR | 160.8116 | 35.79423 | 144.3381 | 39.46656 | 201.1556 | 57.68555 | 192.3714 | 33.17162 |
| TIDS | 401.1884 | 81.17074 | 429.0857 | 87.05238 | 546.0389 | 80.10901 | 523.9429 | 57.18487 |
| TIrr | 136.0721 | 59.86232 | 147.8714 | 56.0894 | 177.6 | 46.08827 | 133.9714 | 61.98684 |

**Typical feature——Low frequency energy**

Appendix Table 5 the feature Low frequency energy comparison for HS HRV, HS DSV and HRV of ECG in four emotions

| Features | Relaxed | | Happy | | Sad | | Angry | |
| --- | --- | --- | --- | --- | --- | --- | --- | --- |
|  | average | std | average | std | average | std | average | std |
| aLF of HS HRV/ms^2 | 452.1098 | 296.7454 | 687.5743 | 369.8149 | 1058.825 | 527.6118 | 892.9007 | 533.7433 |
| aLF of HS DSV/ms^2 | 3813.148 | 2221.562 | 6132.422 | 2023.034 | 9103.065 | 3242.944 | 10323.84 | 4319.649 |
| aLF of ECG HRV/ms^2 | 471.0170 | 355.8666 | 751.2519 | 383.1226 | 1006.189 | 509.6005 | 919.14 | 581.8442 |

**Typical feature——Approximate entropy**

Appendix Table 6 the feature Approximate entropy comparison for HS HRV, HS DSV and HRV of ECG in four emotions

| Features | Relaxed | | Happy | | Sad | | Angry | |
| --- | --- | --- | --- | --- | --- | --- | --- | --- |
|  | average | std | average | std | average | std | average | std |
| ApEn of HS HRV | 0.15034 | 0.03159 | 0.14490 | 0.03969 | 0.09576 | 0.02649 | 0.12004 | 0.02732 |
| ApEn of HS DSV | 0.15005 | 0.02519 | 0.13939 | 0.02832 | 0.10738 | 0.02337 | 0.12177 | 0.01686 |
| ApEn of ECG HRV | 0.14803 | 0.03020 | 0.13637 | 0.03752 | 0.094 | 0.03442 | 0.11049 | 0.02864 |

**Typical feature——DFA in short term**

Appendix Table 7 the feature DFA in short term comparison for HS HRV, HS DSV and HRV of ECG in four emotions

| Features | Relaxed | | Happy | | Sad | | Angry | |
| --- | --- | --- | --- | --- | --- | --- | --- | --- |
|  | average | std | average | std | average | std | average | std |
| $\alpha1$ of HS HRV | 0.83621 | 0.21317 | 1.05062 | 0.24993 | 1.2550 | 0.18160 | 1.14865 | 0.17509 |
| $\alpha1$ of HS DSV | 0.95444 | 0.17491 | 1.04256 | 0.15247 | 1.26529 | 0.11973 | 1.16566 | 0.10108 |
| $\alpha1$ of ECG HRV | 0.83681 | 0.21473 | 1.01048 | 0.21078 | 1.26789 | 0.23183 | 1.20720 | 0.20594 |

1. [↑](#footnote-ref-1)
